# Mitochondrial ATP-Dependent Proteases—Biological Function and Potential Anti-Cancer Targets

**DOI:** 10.3390/cancers13092020

**Published:** 2021-04-22

**Authors:** Yue Feng, Kazem Nouri, Aaron D. Schimmer

**Affiliations:** 1 Princess Margaret Cancer Centre, University Health Network, Toronto, ON M5G 1L7, Canada; yue.feng@mail.utoronto.ca (Y.F.); Kazem.nouri@uhnresearch.ca (K.N.); 2 Department of Medical Biophysics, University of Toronto, Toronto, ON M5G 1L7, Canada

**Keywords:** mitochondria, cancer, protease, ClpXP, AML

## Abstract

**Simple Summary:**

Alterations of cellular metabolism and bioenergetics, oxidative stress, and intracellular reactive oxygen species (ROS) levels are hallmarks of cancer development. Mitochondrial proteases, especially ATP-dependent proteases are essential to regulate mitochondrial function by maintaining protein quality. Emerging studies suggest the therapeutic potential of targeting the matrix ATP-dependent protease ClpXP for a subset of malignancies. In this review, we summarize our current knowledge on the biological function and the anticancer effects of targeting ATP-dependent proteases with a focus on ClpXP.

**Abstract:**

Cells must eliminate excess or damaged proteins to maintain protein homeostasis. To ensure protein homeostasis in the cytoplasm, cells rely on the ubiquitin-proteasome system and autophagy. In the mitochondria, protein homeostasis is regulated by mitochondria proteases, including four core ATP-dependent proteases, m-AAA, i-AAA, LonP, and ClpXP, located in the mitochondrial membrane and matrix. This review will discuss the function of mitochondrial proteases, with a focus on ClpXP as a novel therapeutic target for the treatment of malignancy. ClpXP maintains the integrity of the mitochondrial respiratory chain and regulates metabolism by degrading damaged and misfolded mitochondrial proteins. Inhibiting ClpXP genetically or chemically impairs oxidative phosphorylation and is toxic to malignant cells with high ClpXP expression. Likewise, hyperactivating the protease leads to increased degradation of ClpXP substrates and kills cancer cells. Thus, targeting ClpXP through inhibition or hyperactivation may be novel approaches for patients with malignancy.

## 1. Introduction

Mitochondria are vital membrane-bound organelles in the cytoplasm of almost all eukaryotic cells [1]. These organelles are oxygen-consuming electrochemical generators, where, in the process of oxidative phosphorylation (OXPHOS), the reduction of O_2_ is electrochemically coupled to the conservation of energy in the form of ATP [2,3]. Mitochondria are a biochemical hub where more than 1000 biochemical reactions occur. These reactions go far beyond the oxygen-dependent production of ATP and generate metabolites, macromolecules for nucleotide and protein synthesis, and reactive oxygen species. Mitochondria also regulate a broad spectrum of biological functions including cellular chemotaxis, stress responses, cell signaling, regulation of apoptosis, and development [4,5]. Given their vital role in modulating many cellular functions, it is not surprising that mitochondrial dysfunction contributes to the pathophysiology of many diseases ranging from neurodegenerative disorders and heart failure to diabetes and cancer.

Given the important role of mitochondria, maintaining proper protein homeostasis in the organelle is critical, as is the ability to isolate and eliminate damaged mitochondria [4]. Mitochondrial proteostasis is mediated by specialized molecular chaperones and proteases to maintain mitochondrial protein homeostasis [6,7,8].

Mitochondrial proteases (i.e., mitoproteases) are the primary regulators of mitochondrial proteostasis. Initially, mitoproteases were thought to primarily function as quality control enzymes that removed damaged proteins from sub compartments of the mitochondria. Upon further study, however, additional roles for these proteases have been identified. It is now understood that mitoproteases also process mitochondrial proteins into mature and functional forms, regulate protein half-life, and, in some cases, act as scaffolds independent of their proteolytic activity [7].

Mitochondria harbor a proteome of more than 1000 proteins, containing at least 45 proteases distributed in the outer membrane (OM), intermembrane space (IMS), inner membrane (IM), or mitochondrial matrix [6,9]. These proteases are encoded in the nucleus, synthesized in the cytoplasm, and are subsequently transferred into the mitochondria through a series of import channels and carriers [5,10].

Few of the mitochondria-localized enzymes are called pseudomitoproteases, which are catalytically inactive but functionally proficient as subunits of proteolytic complexes. The remaining mitoproteases are divided into functional categories such as mitochondrial processing protease (MPP), oligopeptidases, ATP-dependent proteases, and other mitochondrial peptidases [6,7,8,9] (Table 1).

Mitochondrial processing peptidases are mostly localized to the matrix and are among the first identified mitoproteases [5,11]. Many mitochondrial precursor proteins require proteolytic removal of their mitochondrial targeting sequences (MTSs) once imported into the mitochondrial matrix. The mitochondrial processing peptidase MPP, which consists of two subunits, the proteolytic subunit PMPCB and the catalytically inactive subunit PMPCA, cleaves MTSs in the mitochondrial matrix. Protein processing is not limited to the matrix. Some proteins also require further processing for their stabilization by the mitochondrial intermediate peptidase (MIP), inner membrane protease (IMMP), or in the intermembrane space (IMS). Proteases from this family, that are involved in these processes, include the presenilin-associated rhomboid-like protease (PARL), the IM metalloendopeptidase OMA1, Met aminopeptidase 1D (METAP1D), and the X-Pro aminopeptidase 3 (XNPEP3) [4,5]. For example, upon loss of mitochondrial membrane potential, OMA1 cleaves the inner mitochondrial protein OPA1 to regulate mitochondrial dynamics [12].

**Table 1:** Intrinsic mitochondrial proteases, functions, and related diseases.

Category	Protease	Mitochondria Localization	Function	Pathology	References
ATP-dependent proteases	YME1L (i-AAA)	IM/IMS	Mitochondrial biogenesis PQC Lipid trafficking Protein import	Optic atrophy Pancreatic ductal adenocarcinoma	[12,13,14,15,16]
	AFG3L2 AFG3L2/SPG7 (m-AAA)	Matrix/IM	Mitochondrial biogenesis PQC MCU assembly	Spinocerebellar ataxia Spastic ataxia 5	[5,17,18,19,20,21]
	CLPP	Matrix	Mitochondrial biogenesis PQC	Acute myeloid leukemia Prostate cancer Breast cancer Lung cancer Liver cancer Ovarian cancer Bladder cancer Thyroid cancer Perrault syndrome 3	[17,22,23,24,25,26,27,28,29]
	LONP1	Matrix	Mitochondrial biogenesis PQC Hypoxia adaptation	Non-small-cell lung cancer Cervical cancer Bladder cancer Colon cancer Acute myeloid leukemia Glioma Lymphoma CODAS syndrome	[17,23,30,31,32,33,34,35,36,37,38,39,40,41]
Processing peptidases	ATP23	IMS	processing peptidase PQC F1FO-ATP synthase assembly	Unknown	[42,43]
	IMMP1L IMMP2L	IM/IMS	Protein maturation Apoptosis/senescence	Thyroid cancer Gilles de la Tourette syndrome Aniridia	[44,45,46,47,48,49]
	OMA1	IM/IMS	Mitochondrial dynamics Mitophagy and apoptosis	Gynecological cancer Breast cancer Colorectal cancer	[12,17,50,51,52]
	PARL	IM	Mitophagy and apoptosis Lipid trafficking Coenzyme Q biosynthesis Complex III assembly	Parkinson disease Striatal neuronal injury Type 2 diabetes mellitus	[53,54,55,56,57,58,59]
	METAP1D	Matrix	Protein maturation	Colon cancer	[5,17,60,61]
	MIP	Matrix	Coenzyme Q biosynthesis Complex III and IV activity Protein import and activation	Left ventricular non-compaction, hypotonia, and infantile death	[5,62,63,64]
	PMPCB	Matrix	Protein maturation	Cerebellar atrophy	[5,65,66]
	XPNPEP3	Matrix	Protein maturation Protein stability	Nephronophthisis-like nephrop-athy	[67,68]
Oligopeptidases	MEP	IMS	PQC	Acute myeloid leukemia	[5,17,69]
	PITRM1	Matrix	PQC	Amyloidotic neurodegeneration	[5,17,70,71]
Pseudoproteases	PARK7	Matrix	PQC Mitochondria dynamics Mitophagy and apoptosis Hypoxia adaption	Parkinson disease Astrocytoma Breast cancer Ovarian cancer Prostate caner	[72,73,74,75,76,77,78,79,80,81]
	PMPCA	Matrix	Protein import and activation	Cerebellar ataxias	[82,83]
	UQCRC1	IM	Oxidative phosphorylation	Rett syndrome, Obesity Breast/ovarian cancer Pancreatic cancer Kidney cancer	[84,85,86,87,88,89]
	UQCRC2	IM	Oxidative phosphorylation	Metabolic Decompensation Colorectal cancer	[84,90,91,92]
	PRSS35	Unknown	Oocyte fertilization	Nonsyndromic cleft lip and palate Kidney fibrosis Squamous cell carcinomas	[52,93,94,95]
Other mitochondrial proteases	HTRA2 (OMI)	IMS	PQC Mitophagy and apoptosis Stress signaling	Ovarian serous carcinomas Parkinson disease and essential tremor	[19,96,97,98,99,100]
	LACTB	IMS	Mitochondrial biogenesis PE metabolism	Breast cancer Colorectal cancer Liver cancer	[17,101,102,103,104]

Abbreviations: IM, inner membrane; IMS, intermembrane space; PQC, protein quality control; MCU, mitochondrial Ca^2+^ uniporter; CODAS syndrome, Cerebral, ocular, dental, auricular, and skeletal syndrome; PE, phosphatidylethanolamine.

**Figure 1:**
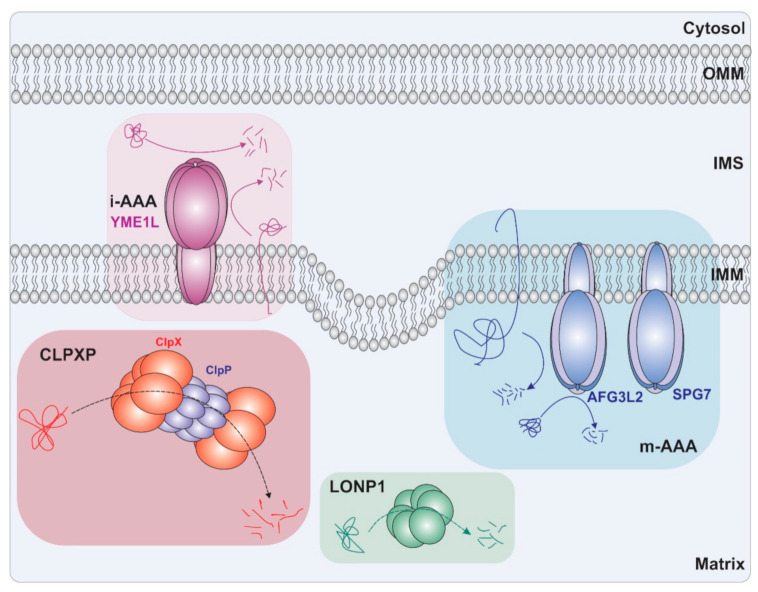
Schematic representation of mitochondrial ATP-dependent proteases. Mammalian mitochondria comprise four different proteases of the AAA+ superfamily for regulating protein quality control: The ClpXP complex and Lon protease 1 in the matrix and the i-AAA and m-AAA proteases in IM. Abbreviations: IMM, inner mitochondrial membrane, OMM, outer mitochondrial membrane; IMS: intermembrane space.

The ATP-dependent proteases, which are the focus of this current review, are found in all mitochondrial compartments. These multimeric, cylinder-shaped complexes, mediate the active remodeling, unfolding, and degradation of mitochondrial proteins using energy derived from ATP hydrolysis. The four known members of this class include the mitochondrial ATP-dependent protease La (Lon) localized to the mitochondrial matrix, the integral membrane proteases i-AAA, and m-AAA proteases localized to the inner mitochondrial membrane, and the ClpXP complex in the mitochondrial matrix (ClpXP: the serine protease ClpP and the AAA+ ATPase ClpX) [5,9] (Figure 1).

Oligopeptidases are present in both the matrix (e.g., PITRM1) and the IMS (e.g., MEP). They degrade the proteolytic products of ATP-dependent proteases as well as mitochondrial signaling sequences cleaved off from new imported mitochondrial proteins [5].

Lastly, other mitoproteases, such as LACTB (β-lactamase-like protein), and HTRA2 (high temperature requirement mitochondrial serine protease A2) in intermembrane space (IMS), also act as regulators of mitochondrial lipid metabolism [104] and play a critical role in maintaining mitochondrial cristae structure [100], respectively.

In this review, we will discuss the ATP-dependent proteases with a primary focus on the ClpXP. We will discuss the biological function of ClpXP and the evidence supporting the development of ClpXP ligands for the treatment of cancer.

## 2. ATP-Dependent Proteases and Their Cellular Function

### 2.1. i-AAA

The i-AAA is a 733-residue integral hexameric membrane metalloprotease encoded by the nuclear YME1L1 gene and located in the inner membrane of mitochondria (IMM) [5,105] with both the AAA+ ATPase and proteolytic domains facing the IMS site [5,106] (Figure 1). i-AAA functions in the proteolytic clearance of misfolded proteins in the IMS and IMM [107]. i-AAA recognizes degrons on the N- or C-terminus of its substrates. The recognized substrate then will actively be unfolded and degraded in a processive manner by i-AAA [107]. Furthermore, in response to stimuli such as increased ROS production, dissipation of mitochondrial membrane potential, heat shock, and loss of mitochondrial DNA (mtDNA), this enzyme performs central regulatory roles by complete or partial proteolysis of specific proteins, including OPA1 (optic atrophy 1), TIMM17A (translocase of inner mitochondrial membrane 17A) and ROMO1 (Reactive oxygen species modulator 1). For example, YME1L1, together with the OMA1 process, OPA1; hence, balancing mitochondrial fusion and fission [5]. In addition, under stress conditions such as changes in levels of ATP, i-AAA and the OMA1 metalloprotease participate in reciprocal proteolysis causing mitochondrial depolarization [5,108]. i-AAA also has a direct effect on the morphology of mitochondrial cristae and the turnover rates of NDUFB6 (NADH dehydrogenase [ubiquinone] 1 beta subcomplex subunit 6) and ND1 (NADH-ubiquinone oxidoreductase chain 1) subunits of Complex I and COX4 (Cytochrome c oxidase subunit 4) from mitochondrial Complex IV [105]. i-AAA inhibition results in the accumulation of these respiratory subunits and consequently increased oxidative stress, which leads to reduced cellular proliferation [105]. Moreover, i-AAA mediates the turnover of PRELID1 (protein of relevant evolutionary and lymphoid interest 1) [15], which facilitates the accumulation of cardiolipin in mitochondrial membranes and modulates apoptosis [14].

### 2.2. m-AAA

m-AAA protease is an integral membrane metalloprotease encoded by the AFG3L2 gene that preferentially localizes to the inner membrane and exposes its catalytic activity toward the matrix site [109] (Figure 1). This protease is formed by homo-oligomers of AFG3L2 (AFG3 ATPase family gene 3-like 2) or hetero-oligomers with AFG3L2 and paraplegin, a homolog of m-AAA encoded by the nuclear SPG7 gene, which is co-localized to IMM and shares the same topology [4,5]. Both m-AAA and paraplegin play a crucial role in the maintenance of aerobic respiration by regulating mitochondrial protein quality and degradation. The reduction of these proteases leads to a variety of respiratory deficiencies and causes deleterious cellular phenotypes such as Complex I deficiency and increased cellular sensitivity to oxidative stress [110]. m-AAA also implements vital functions which are essential in neuronal cells and mutations in AFG3L2 and SPG7 result in neurodegenerative phenotypes [4,18,111,112]. Furthermore, the m-AAA protease proteolytically processes key substrates and thereby regulates mitochondrial protein synthesis and network integrity [113,114].

### 2.3. Lon Protease (LonP)

LonP1 is a 959-residue long AAA+ serine protease that resides in the mitochondrial matrix and forms homohexameric complexes (Figure 1). It is encoded by the nuclear LONP1 gene [36]. The primary function of this ATP-dependent serine protease is to maintain the mitochondrial proteome, mainly via the clearance of misfolded, unassembled, or oxidatively damaged proteins [115,116]. As part of the cell’s adaptation to stress or changes in respiratory conditions, LonP1 also targets specific subunits of respiratory complexes for degradation. For example, LonP1 directly interacts and degrades peripheral arm subunits of complex I [117], facilitates the turnover of SDHFA2 in complex II [118], and degrades isoform 1 of the cytochrome c oxidase subunit 4 (COX4-1) [119]. LonP1 also regulates metabolic pathways such as renal glutamine catabolism [120], suppresses the transfer of cholesterol from the outer mitochondrial membrane to the inner mitochondrial membrane [121], and participates in heme biogenesis [122,123]. LonP1 is also involved in the regulation of mitochondrial DNA (mtDNA) copy numbers and mitochondrial transcription by selective downregulation of mitochondrial transcription factor A (TFAM), a DNA binding protein required for mtDNA maintenance [124,125].

### 2.4. ClpXP

ClpP is a 277 amino acids protease that resides in the mitochondrial matrix of a wide variety of eukaryotes, including humans. It is encoded on chromosome 19 [126]. After translation in the cytosol, it translocates to the mitochondrial matrix by a mitochondrial targeting sequence (MTS), which is cleaved upon protein maturation in the matrix [127,128].

Mitochondrial ClpP is comprised of two identical stable heptameric rings which form a large cylindrical tetradecamer with an aqueous chamber containing 14 internal catalytic cleavage sites [9,129]. While bacterial ClpP mostly exists as a double-ring tetradecamer, mitochondrial ClpP exists as an inactive but stable single heptamer ring. It exists in extended, compacted, and compressed conformational states but only the extended form is catalytically active and degrades substrates [127,129,130,131].

Mitochondrial ClpP lacks ATPase activity and each subunit digests small peptides up to six amino acids [132]. Mitochondrial ClpP complexes with its ATPase (ClpX) to form an active protease with processive proteolytic activity to degrade full-length substrates [113,127,129].

ClpX is the only known ATPase component for mammalian ClpP. It is a nuclear-encoded protein and a member of ATPases associated with various cellular activities (AAA+) superfamily [129]. Similar to ClpP, ClpX also has an N-terminal mitochondrial targeting sequence (MTS). It is a hexameric ring with 6-fold symmetry and becomes stable by binding to ATP [127].

To form the ClpXP protease complex, each end of the barrel-shaped ClpP tetradecamer is capped with the ClpX hexamer [133] (Figure 1). This interaction is mediated by interactions between the flexible N-terminal loop of ClpP and the pore-2 loop of ClpX and is stabilized by a tripeptide IGF loop on ClpX [9,133,134,135].

In an ATP-dependent process, ClpX recognizes and unfolds substrates for degradation and then feeds them into the lumen of ClpP’s proteolytic chamber where they are degraded into small peptides fragments independent of ATP hydrolysis [136]. In bacteria, substrate recognition typically depends on specific linear sequence motifs named degrons [135,137,138], but the substrate recognition features of mitochondrial ClpXP are unknown and need further functional characterization.

The main role of mitochondrial ClpXP is to maintain protein quality control by degrading damaged or misfolded substrates such as those involved in electron transport, metabolic processes, and the Citric acid (TCA) cycle [24,26,128,139]. Beyond its role in mitochondrial protein degradation, ClpX also plays a critical role in regulating heme biosynthesis [140], mtDNA nucleoid distribution [141], and promoting apoptosis [141]. Studies in *Caenorhabditis elegans* also demonstrate that ClpXP regulates the mitochondrial unfolded protein response (UPRmt). In *Caenorhabditis elegans,* small peptides generated by ClpXP-mediated protein degradation upon mitochondrial stress are exported out of the mitochondria into the cytoplasm by the HAF-1 transporter and initiates the mtUPR response in the nucleus [1,142,143]. While ClpXP is an important regulator of mtUPR in the worm, additional studies are needed to clarify the role of this protease in mtUPR in mammalian cells as ClpP inhibition in AML did not alter the expression of mtUPR proteins [24].

## 3. ATP-Dependent Proteases and Cancer

ATP-dependent proteases (AAA+ proteases) play critical roles in the clearance of misfolded or damaged proteins from the mitochondria. Given that changes in cellular metabolism and bioenergetics, oxidative stress, and intracellular ROS level are hallmarks of cancer development, these proteases are also important for the proliferation and metastasis of certain cancers. Therefore, targeting AAA+ proteases might have efficacy against malignancy.

### 3.1. i-AAA, m-AAA and LonP

The link between i-AAA and m-AAA and cancer is not well established or available data are limited. However, LonP1 is expressed at high levels in specific cancers such as cervical, lung, bladder, colon cancer, and acute myeloid leukemia [36,37,38,39,40,41].

Several studies report the effect of LonP1 knockdown in human cell lines. For example, LonP1 knockdown in human lung fibroblasts and bladder cancer cells causes caspase-3-dependent cell death and suppresses cell proliferation [144,145]. LonP1 knockdown also reduces cell proliferation in human mantle cell lymphoma and non-small-cell lung cancer (NSCLC) cell lines [34,35]. Similarly, siRNA knockdown of LonP1 reduces the viability of human malignant glioma cells and radically impairs glioma cell survival under hypoxic conditions [32]. Conversely, LonP1 overexpression promotes cancer cell proliferation, enhances colony formation, and more importantly, increases cellular resistance to apoptosis-inducing reagents [38,146]. Therefore, its inhibition using specific chemical inhibitors has been explored as a potential therapeutic strategy.

For example, triterpenoids such as oleanane 2-cyano-3,12-dioxooleana-1,9-dien-28-oic acid (CDDO), also known as Bardoxolone (RTA-401), and its derivatives such as CDDO-Me inhibit the proteolytic activity of LonP1 [34]. CDDO, in the micromolar range, induces caspase-dependent apoptosis in several human cancer cells with high LonP1 expression, comprising colon carcinoma, B-cell lymphoma, breast ductal carcinoma, and liver hepatocellular carcinoma [34,146]. Phase I clinical trials on CDDO in solid tumors [147] and acute myeloid leukemia [148] have also been conducted but, to date, have failed to confirm the anti-cancer effects in patients.

In addition to CDDO and its derivatives, new classes of LonP1 inhibitors such as γ-lactone Obtusilactone A (OA) have also been discovered and characterized [149]. Further studies show that this natural product inhibits the proteolytic activity of LonP1 both in vitro and in vivo [35]. (−)-Sesamin is another natural product isolated from *Cinnamomum kotoense* that also inhibits LonP1 and induces apoptosis in NSCLC cells [35]. However, the likely toxicity of these inhibitors has limited the interest in developing such compounds.

Inactivating mutations in other mitochondrial proteases leads to developmental, metabolic, and neurological disorders such as CODAS syndrome (CODASS), Perrault syndrome 3 (PRLTS3), optic atrophy 11 (OPA11), erythropoietic protoporphyria (EPP), spinocerebellar ataxia 28 (SCA28), and hereditary spastic paraplegia 7 (HSP7) [20,28,150,151]. Likewise, Lon is essential for survival in mammals, and homozygous deletion of LONP causes early embryonic lethality [152]. Thus, the potential toxicity of inhibiting these proteases as anti-cancer therapies could be high.

### 3.2. ClpXP and Cancer

In contrast to the proteases mentioned above, targeting ClpXP may eliminate malignant cells while sparing normal tissues. In support of this hypothesis, ClpP -/- mice are viable, however, slightly smaller, infertile, and have acquired hearing loss [132]. In humans, there are rare individuals from consanguineous families with homozygous inactivating mutations in ClpP. These individuals are viable but also have acquired hearing loss and infertility [28,153]. Therefore, inhibiting ClpXP may have a therapeutic window in the treatment of malignancy, and we focus the remainder of this review on ClpXP as a potential therapeutic target for cancer therapy.

Emerging evidence suggests that alterations in metabolism and bioenergetics are hallmarks of cancer [154,155]. For a subset of patients, including some with acute myeloid leukemia (AML), chronic myeloid leukemia (CML), pancreatic ductal adenocarcinoma (PDAC), and breast cancer [24,156,157,158,159,160,161], have increased reliance on respiratory function due to limited spare reserve capacity [158], increased TCA cycle substrates [159,162] and low metabolic-plasticity upon OXPHOS inhibition [156,162,163]. In addition, optimal biogenesis and OXPHOS function are crucial for metastatic dissemination as shown with PGC-1α (peroxisome proliferator-activated receptor gamma coactivator 1-alpha) silencing in the breast cancer model [164]. These data highlight the therapeutic potential of targeting OXPHOS function and regulation of ROS production.

Consistent with the essential roles of ClpXP in maintaining OXPHOS function, expression of ClpP has also increased in acute myeloid leukemia [24], as well as some patients with solid tumors such as breast, lung, liver, ovary, bladder, prostate, uterus, stomach, prostate, testis, thyroid and non-small cell lung cancer (NSCLC) [26,27,29]. In addition, increased ClpP expression is correlated with poor prognosis and lower metastasis-free survival in patients with breast adenocarcinoma, uveal melanoma, and lung adenocarcinoma [26].

#### 3.2.1. ClpXP and AML

**Figure 2:**
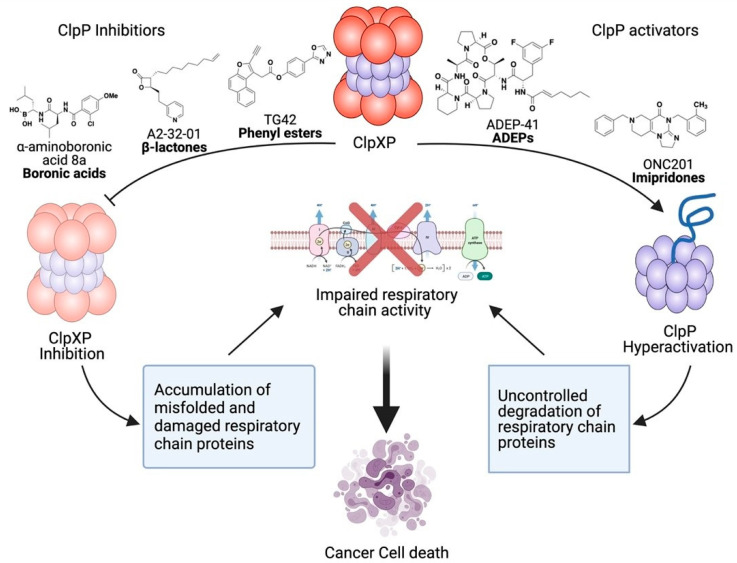
ClpXP inhibition and hyperactivation lead to cancer cell death. Genetic and chemical inhibition of ClpXP leads to the accumulation of misfolded and damaged respiratory chain proteins and impairs oxidative phosphorylation which results in selective cancer cell death. ClpP chemical activators and mutated hyperactive ClpP result in uncontrolled degradation of ClpP substrates. ClpP hyperactivation causes mitochondrial morphological damage and decreased oxidative phosphorylation, which also results in cancer cell death.

Studies support targeting ClpXP for a subset of AML patients. Compared to normal CD34+ hematopoietic cells, 45% of AML patients have increased ClpP expression. Increased ClpP is seen across genetic and molecular subtypes but correlates with increased mitochondrial stress. Genetic and chemical inhibition of ClpP reduces leukemia cell growth and viability and targets leukemic stem cells in vitro and in vivo (Figure 2). Inhibiting ClpP preferentially targets AML cells and primary samples with the highest levels of ClpP while sparing normal hematopoietic cells [24].

Interestingly, hyperactivation of ClpP also effectively targets AML cells by uncontrolled degradation of OXPHOS subunits [25]. Induction of constitutively active ClpP mutant (Y118A) and hyperactivation by imipridones increases degradation of respiratory chain proteins and metabolic enzymes resulting in impaired respiratory function. The small molecule imipridone binds ClpP outside of the active site and produces conformational changes in the enzyme complex resulting in the opening of the axial proteolytic pore and compaction of the protease. Similar changes are produced by the genetic mutant. These changes lead to hyperactivation of ClpP and increase substrate degradation in a selective, but uncontrolled manner. ClpP hyperactivation induces apoptosis in leukemia and lymphoma cells preferentially over normal cells [25]. Thus, these studies support the development of both ClpP inhibitors and activators as novel therapies for malignancy (Figure 2). To date, it is unknown whether inhibiting or activating ClpP is the preferred therapeutic strategy.

#### 3.2.2. ClpXP and Prostate Cancer

ClpXP has also been identified as a novel survivin-associated protein in a proteomic screen using the prostate adenocarcinoma cell line, PC3 [26]. siRNA silencing of ClpP or ClpX in PC3 cells significantly suppresses proliferation and colony formation by decreasing the expression of cyclins A, B1, and D1 and inducing cell cycle arrest. ClpXP is also involved in tumor metastasis and cell migration [26]. PC3 cells with repressed ClpP expression xenografted into immunocompromised mice show decrease invasion and metastasis [26].

#### 3.2.3. ClpXP and Breast Cancer

According to The Cancer Genome Atlas (TCGA) database, ClpP is highly expressed in breast cancer tissue and is correlated with the T stage, ER expression, and lower recurrence-free survival [27]. In addition, loss of ClpP suppresses cell growth, migration, and colony formation in the breast cancer cell lines MDA-MB-231 and ZR-75-1 [27]. In contrast, in the breast adenocarcinoma cell line MCF-7, ClpP is highly expressed but the loss of ClpP had minimal effects on cell proliferation which suggests the importance of ClpP expression in cancer pathology may be cell-type specific [26].

## 4. Therapeutic Development of ClpXP Ligands as Anti-Cancer Agents

### 4.1. ClpXP Inhibitors

#### 4.1.1. ß-Lactones

In 2008, through activity-based protein profiling, trans-ß-lactones were identified that inhibit ClpP by covalently blocking the active site. These compounds have antibacterial effects against *Staphylococcus aureus* (*S. aureus*) [165]. The hydrophobic R1 chain of ß-lactones bind adjacent to the ClpP active site [166] and facilitate its electrophilic core scaffold attacking the catalytic Ser [167]. A synthetic ß-lactone A2-32-01 inhibits recombinant human ClpXP enzyme [24]. In cell culture models, A2-32-01 inhibits AML cell viability and reduces clonogenic growth [24] (Figure 2). In vivo, daily treatment of A2-32-01 reduces leukemic growth without liver, muscle, or renal toxicity [24]. The cyclic ester of ß-lactones is quickly hydrolyzed in human plasma. For example, more than 90% of A2-32-01 is hydrolyzed in cell culture media within 1 h [24]. Thus, although ß-lactones are used as chemical tools to study ClpP inhibition, they are not candidates for clinical development due to their poor selectivity and stability.

#### 4.1.2. Phenyl Esters

Phenyl esters were found through an unbiased high throughput screen of more than 137,000 compounds for their ability to inhibit *S. aureus* ClpP (SaClpP) peptidase activity. Like ß-lactones, phenyl esters also covalently bind to the ClpP catalytic Ser and trap ClpP in the acyl-enzyme intermediate state, which causes deoligomerization of ClpP [168]. Compared to ß-lactones, phenyl ester ClpP inhibitors have improved potency and stability [168]. However, only the AV167 inhibits human ClpP peptidase activity [168].

To improve selectivity for human ClpP, the modified analogues TG42, TG43, and TG53 were generated by substituting a naphtofuran moiety at position-2 [169]. The TG compounds inhibit both hClpP peptidolyic and proteolytic activities [169]. TG42 and TG53 induce apoptosis and inhibit cell migration in Huh7 cancer cells [169] (Figure 2). However, activity-based protein profiling (ABPP) experiments suggest that TG42 interacts with multiple human proteins other than hClpP [169]. Therefore, the mechanism by which TG42 and related compounds induce cell death is uncertain and may be due to effects on targets beyond ClpP. The off and on-target effects of these compounds remain to be elucidated before they can proceed to clinical application.

#### 4.1.3. Boron-Containing Molecules

Boron-containing molecules (BCMs) are another class of compounds that targeting both human and bacterial ClpP. Both C-terminal boronic acids 8a−c (α-amino boronic acids) and N-terminal boronic acids WLS6a inhibit ClpXP enzyme activity in cell-free assays [170,171] (Figure 2). Crystal structure of compound-bound SaClpP and virtual modeling of human ClpP suggest the α-amino boronic acids interact with the catalytic serine residue [171]. Although promising leads for the development of ClpP inhibitors, the stability, potency, and selectivity of these compounds need to be validated and their efficacy in vitro and in vivo needs to be determined.

### 4.2. ClpXP Activators

#### 4.2.1. ADEPs

ADEPs are small molecule ClpP activators that were first isolated from *Streptomyces hawaiiensis* fermentation broth [172]. ADEPs typically contain a lactone core, a phenylalanine linker region, and a hydrophobic tail [173]. These compounds bind ClpP outside of the active site and interrupt the docking of the ClpX IGF loop to ClpP. As a result, these compounds displace ClpX from ClpP. Upon binding to ClpP, these compounds enlarge the ClpP pore leading to selective but uncontrolled proteolysis [173,174] (Figure 2). Originally, ADEPs were discovered as bacterial ClpP activators that inhibit bacterial cell division and kill bacteria by uncontrolled proteolysis [175]. A later study demonstrated that the compounds cross reacts with human ClpP [176]. ADEP-41 kills cancer cell lines including HeLa, U2OS, and undifferentiated SH-SY5Y cell lines [176]. The cytotoxic effects of ADEP are mediated through mitochondrial fragmentation and abolishing OXPHOS function [176].

#### 4.2.2. Imipridones

ONC201, the founding member of imipridones, is in clinical trials for multiple advanced cancers, including hematological malignancies and solid tumors including breast cancer and glioblastoma. Recent studies suggest that other than targeting the bulk cancer cells, imipridones also target cancer stem cells and cancer-associated fibroblasts, as well as activate immune cells in the tumor microenvironment [177,178].

Weekly ONC201 is well tolerated in recurrent glioblastoma patients [179,180]. Early studies suggest that single-agent treatment with ONC201 may prolong survival in a subset of these patients [179]. In addition, combination therapy has also been explored. The addition of exogenous TRAIL receptor agonists prime ONC201 to exert an apoptotic effect in a death receptor (DR5)-dependent manner for non-triple negative breast cancer (TNBC) cells [181]. Also, 2-Deoxyglucose and imipridones result in dual metabolic reprogramming that synergistically depletes energy in glioblastoma [182].

As for the mechanism of action, imipridones, including ONC201, ONC212, and TR compounds were identified as ClpP activators [25,183,184]. Like ADEPs, ONC201 non-covalently binds to the hydrophobic pocket between ClpP subunits and opens the axial entrance [25]. ONC201 treatment abolishes respiratory chain complexes I, II, and IV activity and induces morphological damage of matrix and cristae structure [25,183] (Figure 2). AML, acute lymphoblastic leukemia (ALL) and breast cancer cells with inactive mutant ClpP (D190A) or ClpP knockout are resistant to ONC201 and ONC212, indicating the functional importance of ClpP for imipridones efficacy. Downstream of ClpP activation, imipridones induce the integrated stress response (ISR) and subsequently upregulate the ATF4/CHOP (activating transcription factor 4/CCAAT enhancer-binding protein homologous protein) [25,185].

Nevertheless, ClpP may not be the only target of imipridones. They are also predicted to be dopamine D2 receptor (DRD2) and dopamine D3 receptor (DRD3) antagonists by a machine learning algorithm [186]. Although DRD2 knockout does not abrogate the anti-cancer effect of imipridones, enhanced DRD2/DRD5 heterodimerization was inversely correlated with tumor cell sensitivity to ONC201 [187,188]. Transient DRD2 knockdown also activates the integrated stress response [188].

Therefore, the connection between ONC-induced ClpP activation and dopamine receptor antagonism needs to be determined. In addition, it is unknown which of the downstream molecular effects of ONC201 are related to ClpP activation versus other targets. Finally, how ClpP function and dopamine receptor signaling are involved in ONC resistance is another important field for investigation.

## 5. Conclusions

Given the function of regulating mitochondrial protein quality, ATP-dependent proteases are essential for normal mitochondrial morphology and function. Aberrant activities of ATP-dependent proteases are associated with pathologies ranging from neurodegenerative diseases to cancer [20,32,33,54]. Particularly, emerging studies suggest that LonP1 and ClpXP are needed to alleviate mitochondria stress and promote cancer cell survival and metastasis in a subset of solid and hematology malignancies [24,25,32,34].

Of all the mitochondrial proteases, ClpXP is unique in that humans, animals, and cells remain viable with mild phenotypes despite depletion or mutation of this protease. Coupled with the reported anti-cancer effects of targeting ClpXP, there is a promising therapeutic window to develop clinical-grade ClpXP regulators.

Currently, several classes of inhibitors and activators have been developed to target human ClpP. Mostly, the inhibitors covalently bind to the catalytic serine in ClpP and block ClpP proteolysis activity. In contrast, activators usually allosterically replace ClpX at the ClpP hydrophobic pockets and keep ClpP in the active states for selective but uncontrolled proteolysis. However, the stability, selectivity, and the ability to cross cellular and mitochondrial double membranes remain to be the major obstacles for these molecules to proceed as therapeutic compounds. To date, most efforts have focused on inhibiting ClpP. Fewer studies have focused on developing ClpX targeting molecules. Further studies assessing the efficacy and toxicity of ClpX inhibition in vivo and in vitro are important to evaluate the potential of targeting the ClpX regulatory subunit.

Moreover, considering the overlapping function of ATP-dependent proteases, it is important to understand mechanisms of resistance to ClpXP inhibition and hyperactivation. Moreover, since recent studies suggest ClpXP and LonP1 synergistically regulate cancer cell survival [189], preclinical and clinical studies on combination therapy of ClpXP regulators and other mitoproteases regulators may provide even broader applications of ClpXP regulators in newly-diagnosed and relapsed cancers. Finally, more biological studies are needed to determine the mechanism of action of this protease. For example, while ClpP substrates have been identified, it remains unknown what marks proteins for degradation by this enzyme. These biological studies will provide new insights into mitochondrial biology and help develop ClpXP ligands as novel therapies for subsets of patients with cancer.
